# Acute obstructive cholangitis due to fishbone in the common bile duct: a case report and review of the literature

**DOI:** 10.1186/s12876-019-1088-8

**Published:** 2019-11-07

**Authors:** Min Yu, Bowen Huang, Ye Lin, Yuxue Nie, Zixuan Zhou, Shanshan Liu, Baohua Hou

**Affiliations:** 1 Department of General Surgery, Guangdong Provincial People’s Hospital, Guangdong Academy of Medical Sciences, Guangzhou, 510080 Guangdong China; 2 The Second School of Clinical Medicine, Southern Medical University, Guangzhou, 510000 Guangdong China; 3 Department of Internal Medicine, Peking Union Medical College Hospital, Beijing, 100730 China

**Keywords:** Choledocholithiasis, Common bile duct, Foreign body, Fishbone

## Abstract

**Background:**

Choledocholithiasis is an endemic condition in the world. Although rare, foreign body migration with biliary complications needs to be considered in the differential diagnosis for patients presenting with typical symptoms even many years after cholecystectomy, EPCP, war-wound, foreign body ingestion or any other particular history before. It is of great clinical value as the present review may offer some help when dealing with choledocholithiasis caused by foreign bodies.

**Case presentation:**

We reported a case of choledocholithiasis caused by fishbone from choledochoduodenal anastomosis regurgitation. Moreover, we showed up all the instances of choledocholithiasis caused by foreign bodies published until June 2018 and wrote the world’s first literature review of foreign bodies in the bile duct of 144 cases. The findings from this case suggest that the migration of fishbone can cause various consequences, one of these, as we reported here, is as a core of gallstone and a cause of choledocholithiasis.

**Conclusion:**

The literature review declared the choledocholithiasis caused by foreign bodies prefer the wrinkly and mainly comes from three parts: postoperative complications, foreign body ingestion, and post-war complications such as bullet injury and shrapnel wound. The Jonckheere-Terpstra test indicated the ERCP was currently the treatment of choice. It is a very singular case of choledocholithiasis caused by fishbone, and the present review is the first one concerning choledocholithiasis caused by foreign bodies all over the world.

## Background

The incidence of gallstones is about 15% [1], and the incidence of bile-duct stones accounts for approximately 20% of all gallstones [2]. The calculus in the common bile duct (CBD) may originate from the bile duct system, known as the primary choledocholithiasis. The stones may also have been caused by the decline of stones in the gallbladder, and therefore it is called the secondary choledocholithiasis. The secondary choledocholithiasis is located in the distal of the CBD, which can cause biliary obstruction and infection. Secondary choledocholithiasis is usually considered as an extra-cystic complication of the gallbladder stones, but there are a few exceptions. For example, this paper expounded the case that fishbone entered the CBD through duodenal regurgitation. Choledocholithiasis caused by the foreign body is very rare. There hasn’t been related report about the incidence so far. Choledocholithiasis is mainly manifested as abdominal pain, fever, chills, and jaundice. However, choledocholithiasis caused by the foreign body may present some specific clinical symptoms according to the nature of the foreign body, such as nausea, vomiting, and melena [3, 4]. Primary choledocholithiasis is usually considered to be caused by the translocation of the stones from gall bladder or intrahepatic duct. Nevertheless, patients with secondary choledocholithiasis often have a history of cholecystectomy, EPCP, war-wound, foreign body ingestion, or other particular histories, which need to be paid great attention when diagnosing. The primary treatments for choledocholithiasis are surgery and ERCP currently, and a relatively small proportion of people adopted the methods of conservative treatment [5–7], PTC [8–12] and ESWL [13]. Herein, we report a case of choledocholithiasis caused by fishbone and review all the case reports of choledocholithiasis produced by foreign bodies. A retrospective analysis of the characteristics of the patient population, source of foreign body, clinical manifestation, treatment, and the outcome was conducted. To our knowledge, the present review was the first one concerning choledocholithiasis caused by foreign bodies and may offer some help when dealing with the peculiar secondary choledocholithiasis.

## Case presentation

A 69-year-old Chinese woman with a 6-month history of remittent fever, chilling, jaundice, myalgia, fatigue, and mild headache without abdominal pain was referred to our department. The patient had undergone a BillrothII subtotal gastrectomy for the duodenal ulcer with stenosis 14 years before and cholecystectomy, T-tube choledochostomy and choledochoduodenostomy due to CBD inflammatory stenosis 10 years before. There was no tenderness in her abdomen during admission.

**Fig. 1:**
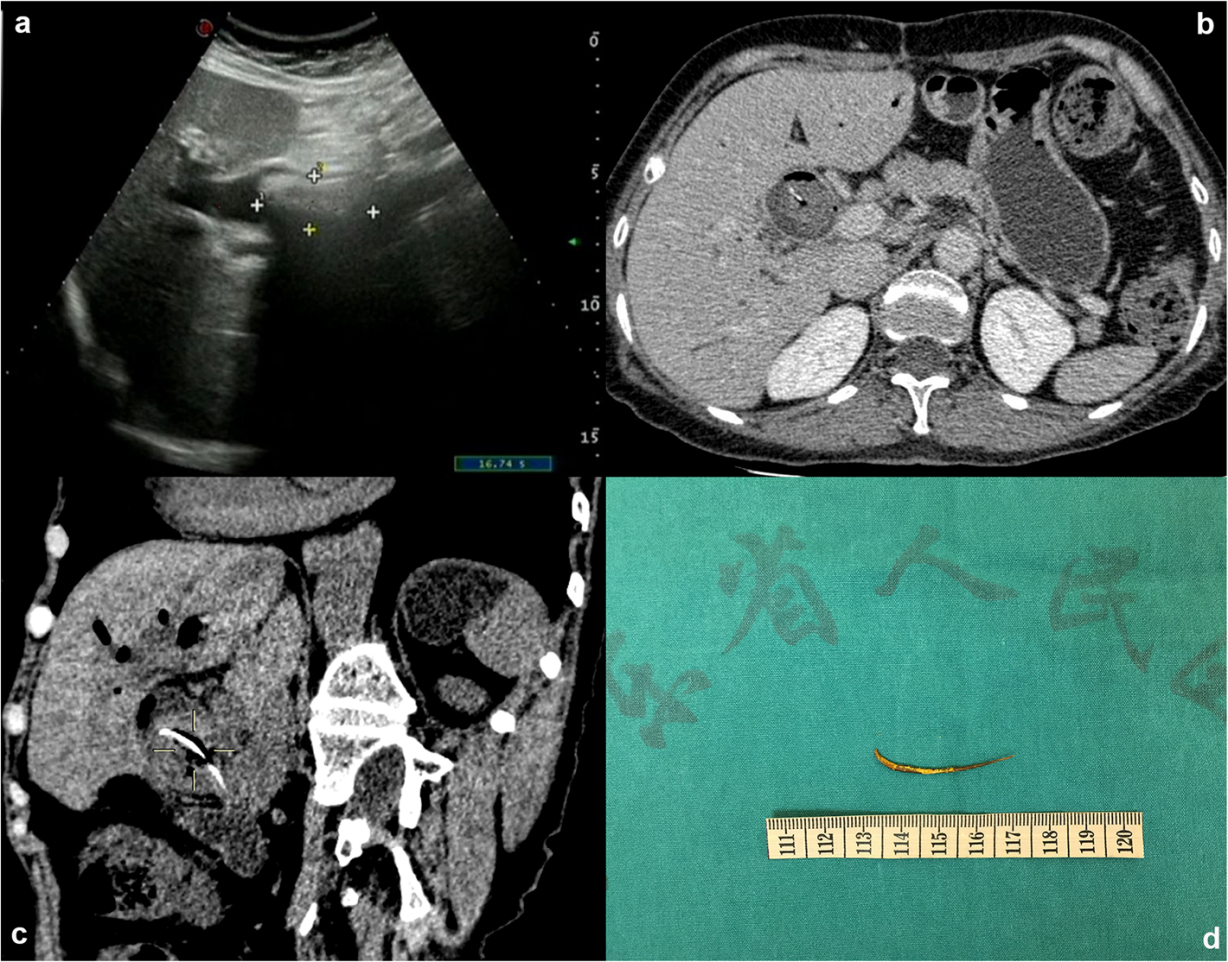
Exploration and truth of the long high-density shadow. **a** Abdominal ultrasonography view of the biliary tract shows choledocholithiasis (4.4 cm × 2.0 cm) with dilatation of intrahepatic and extrahepatic bile duct. **b** Plain CT scan image reveals a strip of hyperdense inside the CBD. **c** Coronal CT view of the long high-density shadow. **d** The photographs of the excised specimen showed a sharp linear fishbone

Blood investigations showed marked impaired liver function of TBIL 19.5 umol/L, ALT 102 U/L and AST 214 U/L. Markers of inflammation were shown to be elevated in patients, such as WBC 15.38 × 10^9/L, NEU% 88.3, procalcitonin (PCT) 4.40 ng/ml (range, 0–0.05). CA19–9 was elevated at 56.52 U/ml (range, 0–27). Ultrasonographic examination of the biliary tract showed choledocholithiasis (4.4 cm × 2.0 cm) with dilatation of intrahepatic and extrahepatic bile duct (Fig. 1a). Of note, the outer layer of the stone was hyperechoic while the inner layer was hypoechoic. The strange phenomenon suggested that calculi may be made of two components at least. Then the patient underwent the upper abdominal enhanced computed tomography (CT), and the results revealed the muddy stone in intrahepatic bile duct with dilatation and pneumatosis and showed post-subtotal gastrectomy feature. However, the most critical finding which CT revealed was a strip of hyperdense inside the CBD, which was 4.0*2.5 cm with CT values about 57HU (Fig. 1b, c). It was worth mentioning that the patient didn’t have any past medical history about stents implantation. We diagnosed choledocholithiasis with acute obstructive cholangitis initially. However, we still didn’t know the essence of the hyperdense hidden in the bile duct.

To prevent the patient from getting worse, we recommended ERC or PTC or surgery as a choice to the patient and her family. As the success ratio of ERC or PTC was decreased due to the large size of the choledocholithiasis and the surgery history, which increased the difficulty of ERC and PTC, the patients and her family chose to perform the surgery. Under general anesthesia, laparoscopic common bile duct exploration (LCBDE) was performed on June 19, 2017. The gallbladder had been removed before, and postoperative adhesion of abdominal cavity was severe. After removed the adhesion, the dilated CBD with a diameter of 2.5 cm was revealed. A small incision was made into the CBD on the upper margin of the duodenum. Intraoperative choledochoscope revealed the massive sandy stone in the CBD.

What’s more, a considerable stone about 4.0 cm × 2.0 cm with irregular shape adhered severely to the adjacent structures. The stone was extracted with a retrieval balloon and basket catheter. When we checked out the calculi removed from the bile duct, unexpectedly the stone was broken down, and we found a fishbone inside. The mass with a strip of hyperdense revealed by CT scan was a fishbone, which migrated into the CBD (Fig. 1d). The patient was uneventful when discharged on the eighth postoperative day, and without recurrence until 21 months after the operation (Additional file 1).

### Literature review

We reported an unusual case of fishbone-induced choledocholithiasis. In this case, the patient’s Oddi sphincter had lost function due to choledochoduodenostomy before, and the fishbone was able to pass through the choledochoduodenal anastomosis and migrate into the CBD. The fishbone acted as a core to form a mixed stone, with cholesterol as its main component ultimately.

It was secluded that the foreign body was hidden in the bile duct. These clinical manifestations always presented a diagnostic dilemma. This case’s only diagnostic clue was linear and sharp calcification within the mass. However, it was hard to connect the linear calcification to the accidentally ingested fishbone because the CBD was isolated from the digestive tract in principle. Thus, identification and removal of the fishbone as soon as possible are essential.

On the other hand, the hidden foreign body in the CBD is rare and can lead to complications which include foreign body related biliary stones. Most cases have been reported as case reports. This study reviews cases of foreign body migration reported in the literature. Method searches and reviews of the literature from “PubMed” search engines using the keywords “foreign body case” and “bile duct” were carried out. Three hundred ninety-seven papers were identified, but details for only 144 cases were available for the present study [3–133]. We specified a protocol for the inclusion of the literature. First of all, the foreign body doesn’t belong to the human body or isn’t a parasite. Secondly, foreign body causes diseases with abnormal migration. Thirdly, the foreign body was hidden in the CBD.

The median age at diagnosed as stones caused by foreign body was 62 years old (range 5 to 91 years). The senior people (range 41 to 80 years) were too fragile to prevent the foreign body from migrating. There was no statistical significance between the genders (the male made up 47.92% versus female 51.39%).

**Fig. 2:**
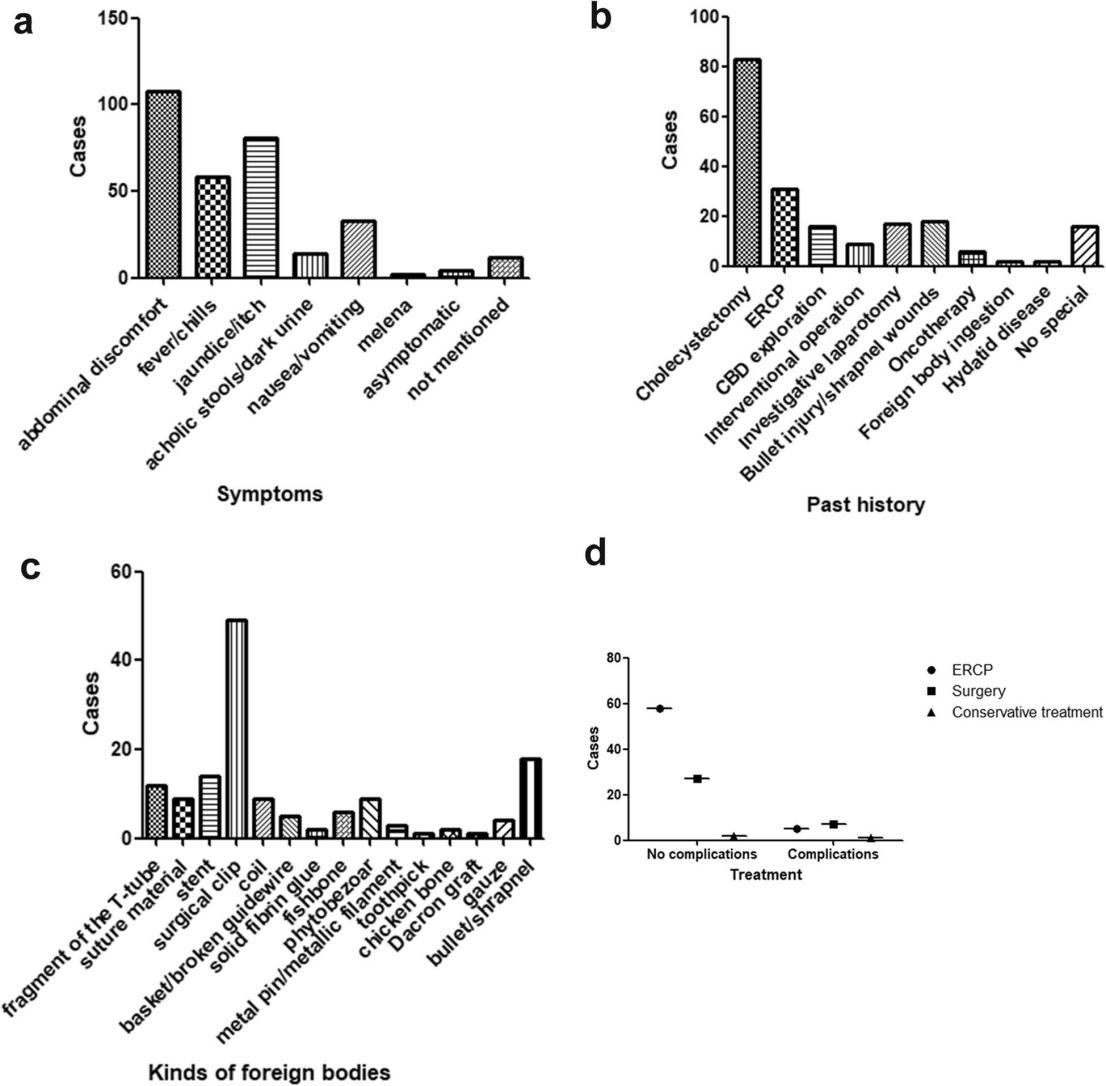
Description of CBD foreign body in the literature review. **a** The different manifestations of foreign body migration patients. **b** The patients’ history. **c** Different kinds of foreign bodies. **d** The different ways to remove the foreign body

Details of the clinical presentations and past medical history were depicted in Table 1. The most common clinical presentations were abdominal pain, fever/chills, and jaundice (Fig. 2a). Most of these patients had suffered cholecystectomy, and ERCP, followed by bullet injury or shrapnel wounds in third place before the foreign body induced choledocholithiasis (Fig. 2b).

**Table 1:** Details of the clinical presentations and past medical history (*n* = 144)

Details	N (%)
Clinical Symptoms	
Abdominal discomfort or abdominal pain	108 (75.0%)
Fever/chills	58 (40.3%)
Jaundice/itch	81 (56.3%)
Acholic stools/dark urine	14 (9.7%)
Nausea/vomiting	33 (22.9%)
Melena	2 (1.4%)
Asymptomatic	4 (2.8%)
Not mentioned	12 (8.3%)
Past Medical History	
Cholecystectomy	83 (57.6%)
ERCP (with sphincterotomy/Stenting)	31 (21.5%)
Common bile duct surgery	16 (11.1%)
Embolization/interventional operation	9 (6.3%)
Investigative laparotomy/abdominal surgery	17 (11.8%)
Bullet injury/shrapnel wounds	18 (12.5%)
Surgery/radiotherapy for carcinoma	6 (4.2%)
Foreign body ingestion	2 (1.4%)
Hydatid disease	2 (1.4%)
No special	16 (11.1%)

There are different kinds of foreign bodies (Fig. 2c). The postoperative complications were the most common cause. The surgical clips (49 accounts for 34.0%), stents (14 accounts for 9.7%) and the fragment of the T-tube (12 accounts for 8.3%) were the most CBD foreign body. There was another kind of foreign body that would pass through the human digestive tract and migrated to the CBD, which included the phytobezoar (9 accounts for 6.3%), fishbone (6 accounts for 4.2%), metal pin (3 accounts for 2.1%), chicken bone (2 accounts for 1.4%)and toothpick (1 accounts for 0.7%). The third significant categories included the debris of bullet or shrapnel (18 accounts for 12.5%).

To choose the best way to remove the foreign body from the CBD, we selected the valid data about the treatment and follow-up. After we made a non-parametric test to compare the outcome about ERCP, surgery and conservative treatment, the Jonckheere-Terpstra test found a significant statistical difference(*P* = 0.044) and indicated the ERCP was the best way to extract the foreign body while the surgery was chasing closely behind (Fig. 2d). Only a relatively small proportion of people used the methods of conservative treatment [5–7], PTC [8–12] and ESWL [13]. The vast majority of victims (92 accounts for 63.89%) recovered uneventfully and were perfectly well at the follow-up clinical examination, but for others the CBD foreign body migration was an omen of misfortune and disaster, it pushed through victims with long-term problems or complications, such as pancreatitis [27], recurrence of cholesterol stones [90, 92], bile leak [90, 99, 127], subhepatic abscess [127], even death [80, 81].

## Discussion and conclusion

Overall, the foreign body migration in the bile duct is rare. However, it is likely that the actual incidence of foreign body migration with resultant biliary complications is underestimated. It’s possible that additional publications, especially in the non-English journals, non-indexed, might have been missed. What’s more, cases of the foreign body hidden in the bile duct might have gone unreported or have been included as part of other types of publications.

In conclusion, although rare, foreign body migration with biliary complications need to be considered in the differential diagnosis for patients presenting with typical symptoms even many years after cholecystectomy, EPCP, war-wound, foreign body ingestion or any other particular history before. The clinical manifestations are similar to that of primary or secondary cholesterol choledocholithiasis, and ERCP is currently the treatment of choice.

This literature review was the first paper concerning on choledocholithiasis caused by foreign bodies and intended to give some suggestions about the differential diagnosis, and the options of treatment for the foreign body migrated to the CBD.

## Supplementary information

**Additional file 1.** Timeline of the case.

## Data Availability

All data generated or analyzed during this study are included in this published article.

## Abbreviations

CBD: Common bile duct
CT: Computer tomography
ERCP: Endoscopic retrograde cholangiopancreatography
ESWL: Extracorporeal shock wave lithotripsy
LCBDE: Laparoscopic common bile duct exploration
PTC: Percutaneous transhepatic cholangio
