# Supplementary material for: Acute obstructive cholangitis due to fishbone in the common bile duct: a case report and review of the literature
Source: BMC Gastroenterol. 2019 Nov 7;19:177. doi: 10.1186/s12876-019-1088-8 (PMC6839197; doi:10.1186/s12876-019-1088-8)
Supplement: Supplementary file 1 — Additional file 1. Timeline of the case. [file 12876_2019_1088_MOESM1_ESM.doc]

CBD inflammatory stenosis

TBIL 19.5 umol/L, ALT 102 U/L, AST 214U/L, WBC 15.38×10^9/L, NEU% 88.3%, procalcitonin(PCT) 4.40ng/ml(range, 0-0.05), CA19-9 56.52 U/ml(range, 0-27)

Ultrasonographic examination of the biliary tract showed choledocholithiasis (4.4cm×2.0cm) with dilatation of intrahepatic and extrahepatic bile duct. Upper abdominal enhanced CT revealed the muddy stone in intrahepatic bile duct with dilatation and pneumatosis and showed post-subtotal gastrectomy presentation

**Laparoscopic common bile duct exploration**

Remittent fever, chilling, jaundice, myalgia, fatigue, and mild headache without abdominal pain

Duodenal ulcer with stenosis

**BillrothⅡ subtotal gastrectomy**

**T-tube choledochostomy and choledochoduodenostomy**

**Discharged**

**Follow-up: without recurrence**

2003 2007 2017.01 2017.06.19 2017.06.27 2019.03
